# Anti-Inflammatory Effect of Extract from *Fragaria ananassa* Duch. Calyx via MAPK and NF-κB Signaling Pathway

**DOI:** 10.4014/jmb.2409.09044

**Published:** 2024-10-31

**Authors:** Hyo-Min Kim, Dan-Hee Yoo, Jung-Wook Kang, In-Chul Lee, Jong-Sup Bae

**Affiliations:** 1 College of Pharmacy, Kyungpook National University, Daegu 41566, Republic of Korea; 2 College of Fusion and Convergence, Seowon University, Cheongju 28674, Republic of Korea; 3 Department of Bio-Cosmetic Science, Seowon University, Cheongju 28674, Republic of Korea

**Keywords:** *Fragaria ananassa* Duch., natural material, anti-inflammatory, MAPK, NF-κB

## Abstract

Currently, *Fragaria ananassa* Duch. are discarded as by-products except for the fruit part, so we developed a natural material using the top (= calyx), one of the by-products, and prepared an extract using 70% ethanol to investigate its effects on anti-inflammatory mechanisms. The polyphenol content of 70% ethanol extracts from *Fragaria ananassa* Duch. calyx was measured to be 265.86 ± 0.85 mg TAE/100 g, respectively. The antioxidant activity was confirmed through the electron donating ability and 2,2'-azino-bis(3-ethylbenzothiazoline-6-sulfonate) (ABTS) radical scavenging ability measurements. When extracts from *Fragaria ananassa* Duch. calyx was treated to LPS-induced RAW 264.7 cells, it was confirmed that the production of inflammation-related factors, NO, PGE_2_, iNOS, COX-2, TNF-a, and IL-6, was inhibited. In addition, it was confirmed that extracts from *Fragaria ananassa* Duch. calyx affected the MAPK signaling pathway by reducing the protein expression of p-ERK, p-JNK, and p-p38, which are the upper signaling pathways. In addition, it was confirmed to reduce the protein expression of p-p65 and p-IκB, which are NF-κB signaling pathways. Therefore, this study suggests that extracts from *Fragaria ananassa* Duch. calyx affect the regulation of the production of major inflammation-related factors by inhibiting the MAPK and NF-κB signaling pathway. These results confirmed that extracts from *Fragaria ananassa* Duch. calyx have the potential to be developed as a new natural material with anti-inflammatory activity.

## Introduction

Inflammation is an immune response to tissue damages caused by infection or harmful stimuli, such as microorganisms and pathogens. It is also referred to as a physiological response within the body and plays an important role in maintaining immune homeostasis [1, 2]. This inflammatory response is regulated by a variety of immune cells, represented by macrophages, which are distributed in the blood and all tissues in the body in the form of monocyte. They play a critical role in the immediate induction and amplification of immunity and inflammation [3, 4]. Macrophages are stimulated by lipopolysaccharide (LPS) existing on the outer membrane of Gram-negative bacterial cells, which is known as a pro-inflammatory substance. This activates Toll-like receptor 4 (TLR4), leading to the activation of extracellular signal-regulated kinases (ERK), p38 kinase, and c-Jun N-terminal kinase (JNK), which are part of the mitogen-activated protein kinase (MAPK) pathway. Additionally, it activates IkappaB (IκB) kinase (IKK), which phosphorylates IκB, resulting in the activation of nuclear factor kappa-light-chain-enhancer of activated B cells (NF-κB) composed of p65 and p50 dimers. [5, 6]. These activated MAPKs and NF-κB are known to increase the production of various pro-inflammatory substances, including inducible nitric oxide synthase (iNOS), cyclooxygenase-2 (COX-2), nitric oxide (NO), and prostaglandin E2 (PGE_2_), and the secretion of pro-inflammatory cytokines such as tumor necrosis factor-alpha (TNF-α), interleukin-6 (IL-6)[7-9]. However, excessive inflammatory responses can lead to autoimmune diseases, rheumatoid arthritis, bronchitis, multiple sclerosis, and cancer, and the development of anti-inflammatory drugs that can control them is of great importance [10, 11]. This has led to the current trend of investigating the efficacy of new natural materials by using studies on the modulation of inflammatory responses to LPS-stimulated macrophages [12, 13].

*Fragaria ananassa* Duch. is a perennial plant that belongs to the Rosaceae family and is a fruit vegetable that is known for its outstanding color, aroma, and its unique combination of sour and sweet flavors. These flavors and aromas make it a popular crop worldwide, and in Korea, the variety called “Seolhyang” is the most commonly cultivated [14-16]. In general, *Fragaria ananassa* Duch. is most commonly consumed as whole fruits or in processed forms such as jelly, jam, ice cream, and juice. As such, only the fruits of *Fragaria ananassa* Duch. are consumed or used, and the stems, leaves, roots, and calyxes are discarded as bio-waste [17]. Utilizing these by-products can reduce the cost of raw materials, and fruits of *Fragaria ananassa* Duch. and by-products are rich in phenolic compounds, which are known to have high antioxidant activity [18]. *Fragaria ananassa* Duch. contains antioxidants such as hydroxybenzoic acid derivatives, anthocyanins, quercetin, flavonoids, polyphenols, and vitamin C. In particular, the flavonoids in *Fragaria ananassa* Duch., such as kaempferol-3-glucoside (astragalin), quercetin 3-glucoside and 3-glucuronide, as well as ellagic acid, procyanidin, and epicatechin, have been reported to have antioxidant, anti-inflammatory, anti-diabetic, anticancer, anti-tumor, and melanin inhibition activities [19-23]. In addition, research findings report that *Fragaria ananassa* Duch. fruit has excellent antioxidant, anti-atherosclerotic, and anticancer properties, as well as anti-inflammatory properties by reducing inflammatory cytokines in macrophages stimulated by LPS [24].

Although many studies have demonstrated the active ingredient analysis and various effects of *Fragaria ananassa* Duch. fruit, there is a lack of research on the by-products that are discarded after consumption of *Fragaria ananassa* Duch. fruit. This study aimed to prepare extracts from *Fragaria ananassa* Duch. grown in Korea using the calyx part, which cannot be consumed or processed, and to verify their anti-inflammatory effects by examining how they affect inflammatory mediators generated through the MAPK and NF-κB signaling pathways.

## Materials and Methods

### Material and sample extraction

**Fig. 1:**
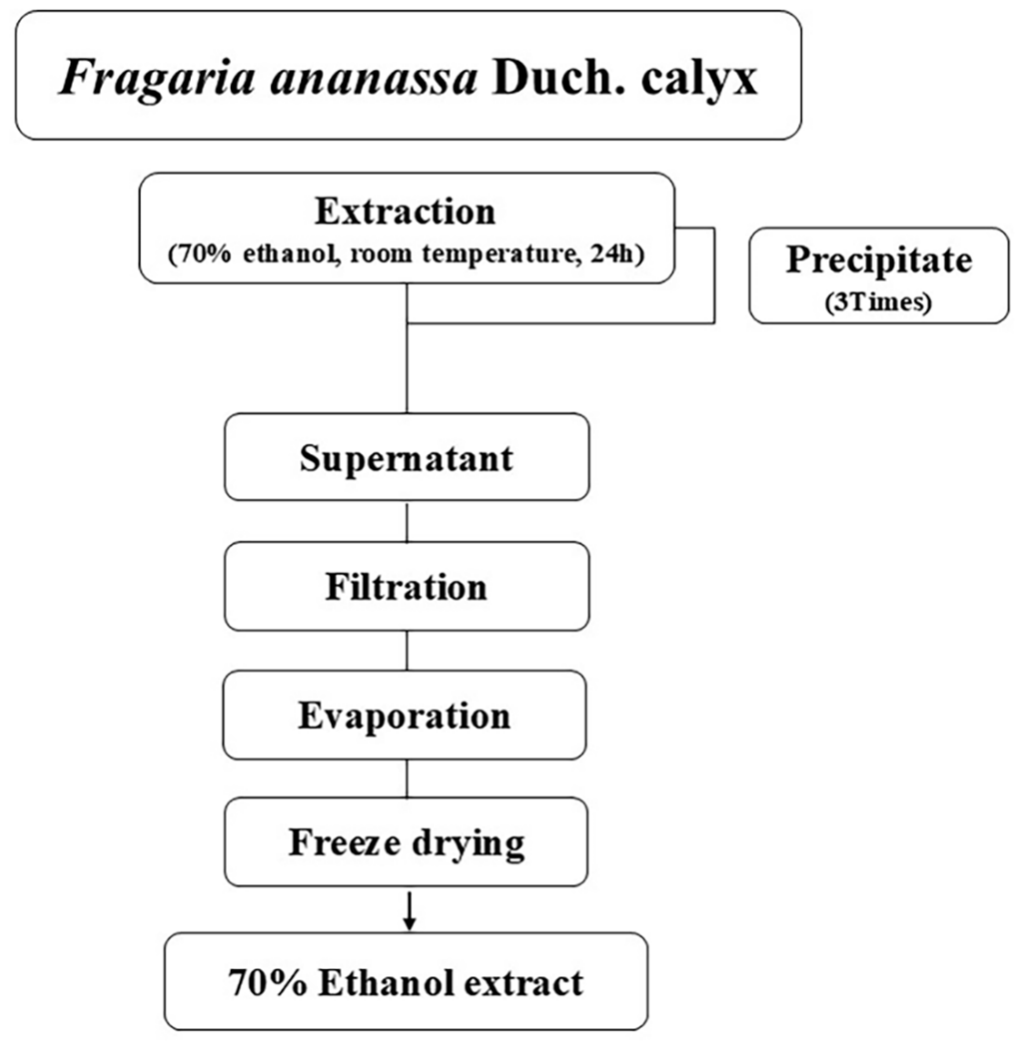
Schematic diagram of extracts from *Fragaria ananassa* Duch. calyx. The *Fragaria ananassa* Duch. calyx was dried and crushed, then soaked in 70% ethanol at room temperature for 24 h. The supernatant and solid material were separated, and the process was repeated two more times. The collected supernatant was filtered, concentrated, and freeze-dried to produce the extract.

*Fragaria ananassa* Duch. calyx used in this experiment were purchased from Jinshiwang Strawberries (Republic of Korea) collected in May. The calyxes were collected after removing all the pulp from the strawberries, and the calyxes were then washed, hot air dried (60°C), and crushed for extraction. Ethanol extraction was performed using 70% ethanol as solvent, with 10 times of the sample weight (g) of 70% ethanol added, stirred (125 rpm, 24 h, room temperature), and then separating the supernatant and precipitate, which was repeated twice. The supernatant completed of extraction was filtered with a vacuum pump and filter paper (Whatman No. 5, No. 4, No. 2), and then the rotary vacuum evaporator (EYELA, Japan) was used for concentration under reduced pressure. The concentrate was adjusted to 15°Brix. After the solvent removal, the extract was lyophilized and stored in powder form at -20°C and used as the sample for this experiment, and the yield of 70% ethanol extracts from *Fragaria ananassa* Duch. calyx was found to be 9.17% (Fig. 1).

### Reagents and Instruments

Folin-ciocalteu reagent (Folin), and tannic acid, the reagents used for the measurement of total polyphenol content, and 1-1-diphenyl-2-picrylhydrazyl (DPPH), and 2,2'-azinobis-bis (3-ethylbenzothiazoline-6-sulfonic acid) (ABTS), the reagents used for the measurement of antioxidant activity, were purchased from Sigma Chemical Co. (USA), and Na_2_CO_3_ from Kanto Chemical Co. (Japan) for use. 3-[4,5-dimethylthiazol]-2-yl]-2,5-diphenyl-tetrazolium bromide (MTT) and dimethyl sulfoxide (DMSO) used for cell viability measurements, and LPS and the Griess reagent used for NO inhibitory activity measurements were purchased from Sigma Chemical Co. for use. The enzyme-linked immunosorbent assay (ELISA) kits used to measure the production of the inflammatory mediators PGE_2_, TNF-α, and IL-6 were purchased from R&D Systems, Inc. (USA). Primary antibodies for β-actin, anti-inflammation-related factors iNOS, COX-2, ERK, JNK, p38, p-65, IkB, p-ERK, p-JNK, p-p38, p-p65 and p-IkB, and secondary antibodies, anti-mouse and anti-rabbit, used in protein expression experiments were purchased from Santa Cruz Biotechnology, Inc. (USA) and Cell Signaling Technology, Inc.(USA) for use. For the enhanced chemiluminescence (ECL) solution, the reagent used to detect protein expression, the Immobilon Western Chemiluminescent HRP Substrate was purchased from Merck Millipore (USA) for use. Trizol reagent used to perform real-time PCR was purchased from Thermo Fisher Scientific (USA) and Go Script Reverse Transcription kits were purchased from Promega (USA). In addition, TaqMan Universal Master Mix and TaqMan Assay (probe) were purchased from Thermo Fisher Scientific for use.

The instruments used in the experiments were a rotary vacuum evaporator (EYELA, Japan), pH meter (STARA2110, Thermo Fisher Scientific), ELISA readers (Molecular Devices, China), CO_2_ incubator (Pantasonic Healthcare Co, Japan), micro refrigerated centrifuge (SPECTROstar Nano, BMG LABTECH, Germany), nano drop (Jcbio, Republic of Korea), SimpliAmp Thermal Cycler (Applied Biosystems, USA), and Chemidoc imaging system (Bio-Rad, USA) .

### Measurement of Total Polyphenol Content

Total polyphenol content was determined by the Folin-Denis method with modifications [25]. The extract was diluted to 100 ppm and used in the experiments and reacted with 50% Folin solution at a ratio of 1:1 for 3 min at room temperature. Then, 10% Na_2_CO_3_ was mixed in an equal ratio and the absorbance (700 nm) was measured after 1 h of reaction at room temperature in a darkroom. A standard curve was plotted using a standard substance (tannic acid), and the content was expressed as mg/ 100 g.

### Measurement of Electron Donating ability (EDA)

The EDA (expressed as DPPH radical scavenging ability) was determined by applying the Blois method [26]. 180 μM DPPH reagent and the extracts diluted by concentration were mixed in a 1:6 ratio and allowed to react for 15 min at room temperature. A microplate reader was then used to measure the absorbance at 517 nm.

### Measurement of ABTS Cationic Radical Scavenging Ability

ABTS cationic radical scavenging ability was measured by the method of Re *et al*. [27] with modifications. 7 mM ABTS was mixed with 2.45 mM K_2_S_2_O_8_and reacted for 18 h to form ABTS cationic radicals. After the reaction was completed, the reagent was diluted with ethanol and used as a solution, and the ABTS solution and the extracts diluted per concentration were added in a 1:1 ratio and measured at absorbance (700 nm).

### Cell lines and Cell Culture

The cell line used in this experiment, macrophage RAW 264.7, was obtained from the Korean Cell Line Bank (KCLB40071), and DMEM containing 10% FBS and 100 U/mL penicillin/streptomycin was used as the cell culture medium and cultured in a 5% CO_2_ incubator at 37°C for cellular stabilization before succession.

### Measurement of Cell Viability (MTT Assay)

Cell viability measurements were performed using the Carmichael method [28]. RAW 264.7 cells were dispensed into a 96-well plate at 1 × 10^4^ cells/well. Extracts were then prepared and added at concentrations of 5, 10, 50, 100, 500, and 1,000 μg/mL, then the cells were stabilized in a 5% CO_2_ incubator at 37°C. Then 40 μL of MTT reagent (2.5 mg/mL) was added to each well and reacted for 4 h. After completing the incubation, the supernatant was removed and 100 μl of DMSO was dispensed, shaken for 10 min at room temperature, and the absorbance (540 nm) was measured using an ELISA reader. Cell viability measurements were expressed as a percentage (%) by comparing the absorbance of the sample-added group to the absorbance of the sample-free group with no sample added as the control group.

### Measurement of NO Production Inhibitory Activity

To measure NO production from RAW 264.7 cells stimulated by LPS, the amount of NO present in the culture medium was measured according to the method of Green *et al*. [29]. RAW 264.7 cells are dispensed at 2 × 10^5^ cells/well into a 6-well plate and stabilized in a 5% CO_2_ incubator at 37°C for 24 h. Cells were then treated with LPS (10 μg/mL) except for the untreated group for induction of NO production, for the LPS single treated group and the sample section. After 2 h, the samples prepared per concentration (50, 100 and 500 μg/mL) were treated and cultured for 18 h. To check NO production, the culture medium and Griess reagent were added in a 1:1 ratio and reacted for 10 min, and absorbance (540 nm) was measured using the ELISA reader. Measurements of NO production inhibitory activity were expressed as a percentage of the non-added group compared to the added group.

### Measurement of PGE_2_ Production

To measure PGE_2_ production, RAW 264.7 cells were used, dispensed at 3 × 10^5^ cells/well in a 6-well plate and stabilized for 24 h. The cells were then treated with the stimulant LPS (10 μg/mL) and allowed to react for 2 h, followed by the addition of *Fragaria ananassa* Duch. calyx 70% ethanol extracts for 18 h. 800 μL of the culture medium was collected and centrifuged at 13,200 rpm (for 5 min) to remove the precipitate to collect the supernatant. The supernatant was reacted with the ELISA kit to determine the production of PGE_2_, and the ELISA kit was run according to the manufacturer's instructions. The production of PGE_2_ was measured by absorbance (450 nm) using the ELISA reader.

### Measurement of Pro-Inflammatory Cytokine (TNF-α, IL-6) Production

To measure the production of the pro-inflammatory cytokines TNF-α and IL-6, RAW 264.7 cells were dispensed at 3×10^5^ cells/well in a 6-well plate and stabilized for 24 h. Inflammation was then induced by treatment with the stimulant LPS (10 μg/mL) for 2 h, followed by the addition of 70% ethanol extract from *Fragaria ananassa* Duch. calyx for 18 h. The productions of TNF-α and IL-6 were determined by centrifuging the culture media at 13,200 rpm and using the supernatant to remove the precipitate. The supernatant was applied to the respective ELISA kits for TNF-α and IL-6, and the experiment was performed according to the method provided by the ELISA kit manufacturer. TNF-α and IL-6 production was measured by absorbance (450 nm) using the ELISA reader.

### Measurement of Protein Expression with a Western Blot

iNOS and COX-2, factors involved in the inflammatory response, were identified using RAW 264.7 cells. RAW 264.7 cells were dispensed at 1 × 10^6^ cells/well in a 100 mm culture dish and incubated for 24 h. Cells were treated with LPS as a stimulus to a concentration of 10 μg/mL, and extracts were processed 2 h later by concentration (50, 100 and 500 μg/mL). After incubation was complete, the medium was removed and washed twice with PBS. Cells were then lysed by preparing lysis buffer with RIPA buffer and Protease & Phosphatase Single-Use Inhibitor Cocktail 100X in a 100:1 ratio and centrifuged at 4°C, 13,200 rpm for 20 min. After centrifugation, the protein in the supernatant was quantified using the BCA protein assay kit and subsequently loaded by electrophoresis on a 10% acrylamide gel. The protein of the gel was then transferred onto a polyvinylidene fluoride (PVDF) membrane using a transfer apparatus and blocked for 1 h at room temperature in 5% skim milk dissolved in tris-buffered saline & tween 20 (TBST). Primary antibody was added and incubated at 4°C overnight, followed by washing with TBST for 10 min, in triplicate. The secondary antibody was then added and reacted for 1 h and 30 min, followed by a 10 min wash with TBST, in triplicate. The bands were then reacted with ECL solution in a darkroom and identified using the ChemiDoc MP Imaging System.

### Total RNA Isolation and cDNA Synthesis

RAW 264.7 cells were dispensed at 1 × 10^6^ cells/well in a 100 mm culture dish and cultured in a 37°C, 5% CO_2_ incubator for 24 h. The stimulant, LPS, was treated to a concentration of 10 μg/mL, followed 2 h later by treating samples per concentration (50, 100, and 500 μg/mL) and incubating for 24 h. The culture medium was then removed, washed twice with PBS, and cells were lysed by adding 1 mL of trizol reagent. 200 μL of chloroform was added to the trizol reagent that lysed the cells, mixed by shaking 10 times, and centrifuged at 4°C, 13,200 rpm for 20 min. After centrifugation was complete, the supernatant was taken and mixed with isopropanol in a 1:1 ratio and shaken 10 times. The supernatant excluding the pallet was then removed by centrifugation at 4°C, 13,200 rpm for 20 min and washed with 1 mL of 75% ethanol diluted with diethyl pyrocarbonate (DEPC) water. After removing all cleaning solution, 50 μL of DEPC water was dispensed and mixed. Total RNA amount was measured using a nano drop. cDNA synthesis was performed by mixing extracted RNA (2 μg) with 1 μL of Oligo (dT) 15 primer (500 mg/mL), adding nuclease free water (NW) and reacting at 75°C (5 min), followed by 4°C (5 min), and then 5X reaction buffer, MgCl_2_, polymerase chain reaction (PCR) nucleotide mix, reverse transcriptase, rnasin inhibitor, and NW were added and reacted at 25°C (5 min), 42°C (60 min), 70°C (15 min), and 4°C (10 min).

### Quantitative Real-Time Polymerase Chain Reaction (qRT-PCR)

To determine the anti-inflammatory effect as mRNA expression using RAW 264.7 cells, the synthesized cDNA was reacted with TaqMan Universal Master Mix and probe. GAPDH, iNOS, and COX-2 were reacted at 50°C (2 min) and 95°C (10 min), then 95°C (15 sec) followed by 40 cycles at 60°C (1 min).

### Statistics Processing

All experiments were independently repeated at least three times, and the same experiment was repeated at least three times. The result values were expressed as the mean ± standard deviation of each item. IBM SPSS Statistics (ver. 23, IBM Corp., USA) was used to conduct a paired-samples *t*-test (*p < 0.05, **p < 0.01 and ***p < 0.001) for significance validation.

## Results

### Measurement of Total Polyphenol Content

The polyphenol content in plants are most often determined using the Folin-Ciocalteús phenol reagent. Polyphenols have antioxidant activity through the scavenging of free radicals and are believed to be protective against diseases such as cardiovascular disease, enzyme inhibition, neurodegenerative diseases, and regulation of carbohydrate metabolism [30, 31].

Tannic acid was used as a standard substance to determine the total polyphenol content of the 70% ethanol extracts from *Fragaria ananassa* Duch. calyx. The result values are expressed as tannic acid equivalent (TAE), the amount of tannic acid contained per 100 grams of extract. The 70% ethanol extracts from *Fragaria ananassa* Duch. calyx showed a polyphenol content of 265.86 ± 0.85 mg TAE/100 g.[Table T1]

### Measurement of EDA (DPPH Assay)

When DPPH reacts with an antioxidant with free radical scavenging ability, DPPHH is formed. The resulting DPPHH has a lower hydrogen content than DPPH and is decolorized, manifesting a yellow color, while DPPH forms diphenyl picrylhydrazine, manifesting a purple color, when combined with a source of hydrogen atoms. This makes DPPH the most readily available method for measuring the potential of antioxidants to reduce free radicals [32, 33].

**Fig. 2:**
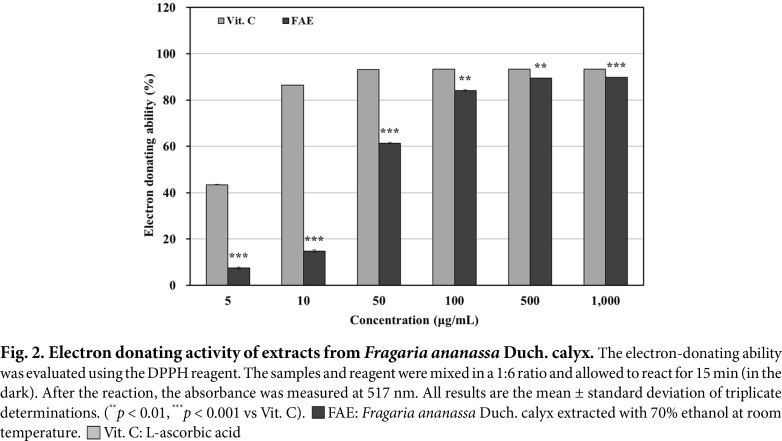
Fig. 2

The results of measuring EDA of 70% ethanol extracts from *Fragaria ananassa* Duch. calyx are shown in Fig. 2. Vitamin C was used as a positive control group, and EDA measurements of 70% ethanol extracts from *Fragaria ananassa* Duch. calyx showed a concentration-dependent increase. At a final concentration of 1,000 μg/mL, the 70% ethanol extracts from *Fragaria ananassa* Duch. calyx showed more than 89% activity (IC_50_ = 48.86 μg/mL), while the positive control group, vitamin C, showed 93.41% activity (IC_50_ = 5.78 μg/mL). This confirmed that the 70% ethanol extracts from *Fragaria ananassa* Duch. calyx and the positive control group, vitamin C, had similar activity at 1,000 μg/mL.

### Measurement of ABTS Radical Scavenging Ability

The ABTS assay is based on the principle that the oxidation of ABTS with the formation of ferrylmyoglobin radicals leads to the formation of ABTS cationic radicals, which are then bleached from blue to colorless through antioxidant reactions [34, 35].

**Fig. 3:**
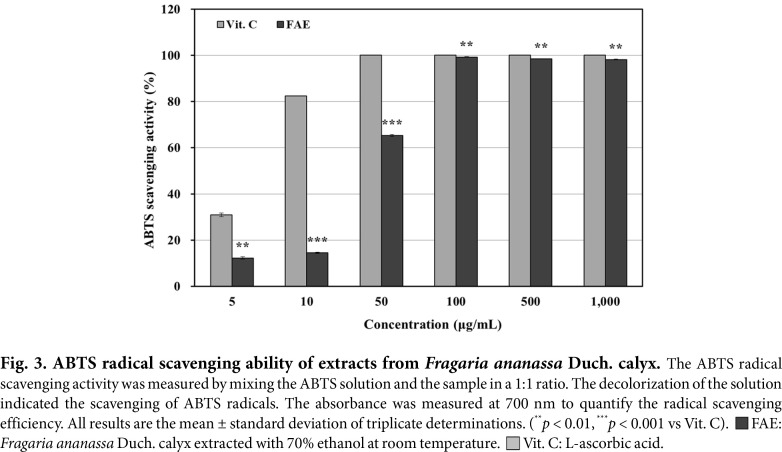
Fig. 3

The 70% ethanol extracts from *Fragaria ananassa* Duch. calyx showed a concentration-dependent increase in activity, which is shown in Fig. 3. At a final concentration of 1,000 μg/mL, the 70% ethanol extracts from *Fragaria ananassa* Duch. calyx exhibited more than 98% antioxidant activity (IC_50_ = 42.88 μg/mL). As a positive control group, vitamin C was used, which showed 100% activity (IC_50_ = 6.48 μg/mL) at 1,000 μg/mL. This confirmed that the 70% ethanol extracts from *Fragaria ananassa* Duch. calyx exhibited similar activity to the positive control group, vitamin C.

### Measurement of Cell Viability (MTT Assay)

A popular method for measuring cell viability is the MTT assay, which utilizes the principle that tetrazolium salts are reduced to insoluble formazan (purple in color) by dehydrogenases in the mitochondria present in living cells. The reduced formazan can then be dissolved in organic solvents such as isopropanol or dimethyl-sulfoxide (DMSO), and the absorbance can be measured [36, 37].

**Fig. 4:**
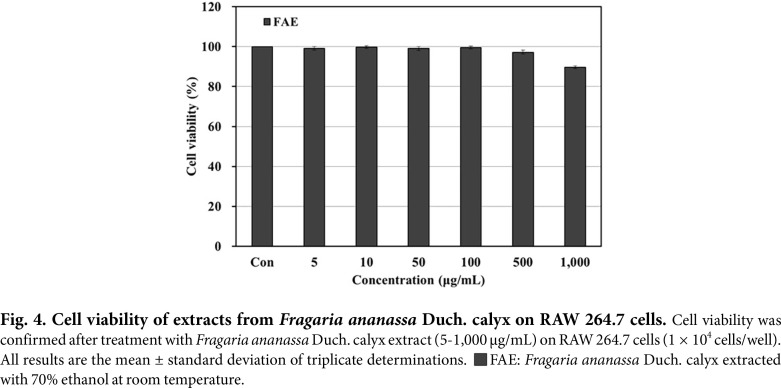
Fig. 4

The MTT assay was performed to determine the cell viability of 70% ethanol extracts from *Fragaria ananassa* Duch. calyx in RAW 264.7 cells, and the results are shown in Fig. 4. The 70% ethanol extracts from *Fragaria ananassa* Duch. calyx demonstrated more than 95% cell viability at 500 μg/mL and below, so subsequent cell experiments were conducted at the concentration range that resulted in more than 95% cell viability.

### Measurement of NO Production Inhibitory Activity

When macrophages are stimulated by LPS, one of the components of the outer membrane of Gram-negative bacteria cells, they produce inflammatory mediators such as NO. NO is known to play a crucial role in a variety of biological processes, including cell death, immune defense, and neurotransmission. This NO is produced by the action of NOS on L-arginine and is produced in large amounts, especially under the influence of iNOS, a type of NOS produced by LPS. This large amount of NO can cause chronic inflammation by inducing healthy host cells to die, contributing to inflammatory pathology [38, 39].

**Fig. 5:**
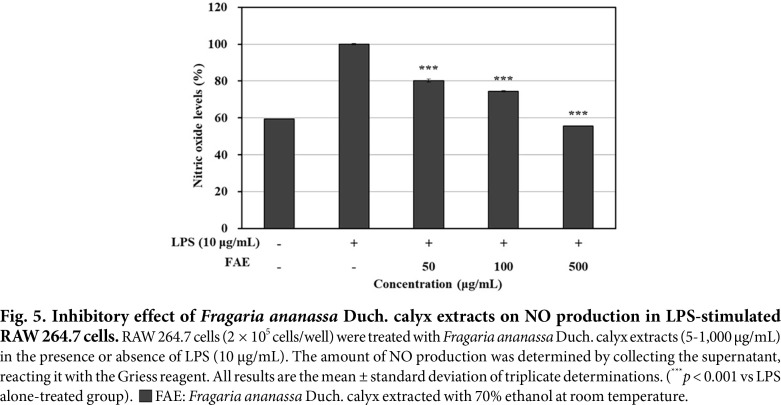
Fig. 5

Experiment was performed by treating with LPS to induce NO production, followed by treatment with 70%ethanol extracts from *Fragaria ananassa* Duch. calyx, and the results are shown in Fig. 5. The difference between LPS single treated group and the untreated group was confirmed, and the 70% ethanol extracts from *Fragaria ananassa* Duch. calyx showed 44.32% NO production inhibitory activity at 500 μg/mL compared to the LPS single treated group.

### Measurement of PGE_2_ Production

In activated inflammatory cells, arachidonic acid causes the synthesis of PGs, including PGE_2_, due to the action of COX-2. The PGE_2_ produced by this inflammatory response is known to be a major inflammatory mediator that causes erythema, tissue reactions, fever and pain due to vasodilation and edema [40, 41].

**Fig. 6:**
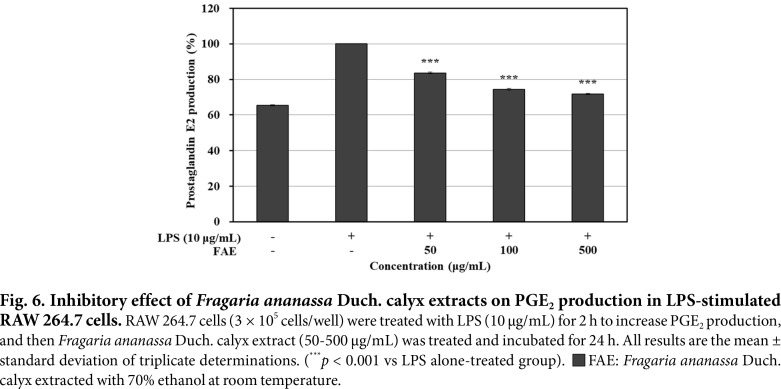
Fig. 6

RAW 264.7 cells with increased PGE_2_ production by LPS stimulation, were treated with 70% ethanol extracts from *Fragaria ananassa* Duch. calyx to measure the inhibitory activity. The results are shown in Fig. 6: the 70%ethanol extracts from *Fragaria ananassa* Duch. calyx at 500 μg/mL showed 28.28% PGE_2_ production inhibitory activity compared to the LPS single treated group.

### Measurement of Pro-Inflammatory Cytokine (TNF-α, IL-6) Production

As one of the important components of the human immune system, macrophages play a critical role in the immune response to foreign substances. TNF-α, IL-6 that occur during this response eliminate foreign substances. However, when pro-inflammatory cytokines are uncontrolled and overexpressed, they cause inflammation, leading to various diseases such as chronic disease degeneration and tissue damage [42, 43].

**Fig. 7:**
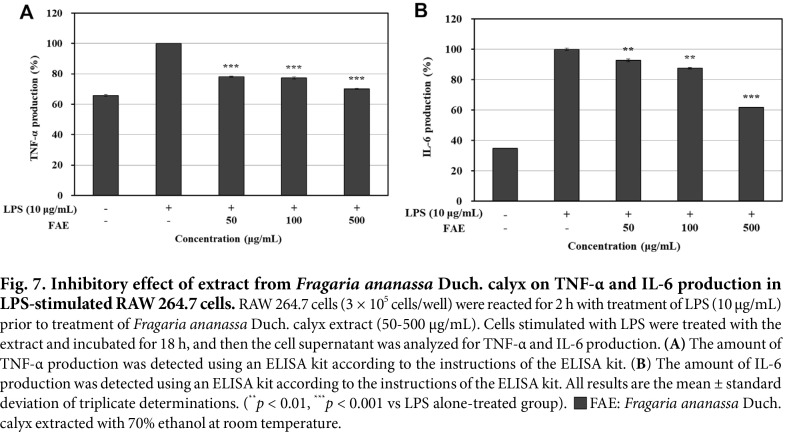
Fig. 7

In this experiment, we investigated TNF-α and IL-6, which are known to be rapidly expressed during the early stages of inflammation among pro-inflammatory cytokines [44, 45]. After stimulating RAW 264.7 cells with LPS to overexpress the production of the pro-inflammatory cytokines TNF-α and IL-6, the inhibitory activity of the 70% ethanol extracts from *Fragaria ananassa* Duch. calyx was identified. The results are shown in Fig. 7A-7B. It was found that the 70% ethanol extracts from *Fragaria ananassa* Duch. calyx inhibited TNF-α production by 29.93% and IL-6 production by 38.42% compared to the LPS single treated group.

### Measurement of the Effect of Protein Expression Inhibition on iNOS and COX-2

Macrophages and hepatocytes, etc. produce iNOS in response to exposure to endotoxins and cytokines. The produced iNOS stimulates the production of NO in large quantity, which plays a key role in the development of various inflammatory diseases. COXs are distinguished by their structure and function, and there are two forms, COX-1 and COX-2. COX-1 is widely present in most cells, and the PGs catalyzed by it are thought to have a protective effect on the gastrointestinal tract. COX-2 is a pathologic enzyme that acts as an enzyme inducing the production of PGs and is believed to be responsible for the development of inflammation [46, 47]. This study sought to confirm its anti-inflammatory activity by determining its inhibitory effects on these inflammation-related factors.

**Fig. 8:**
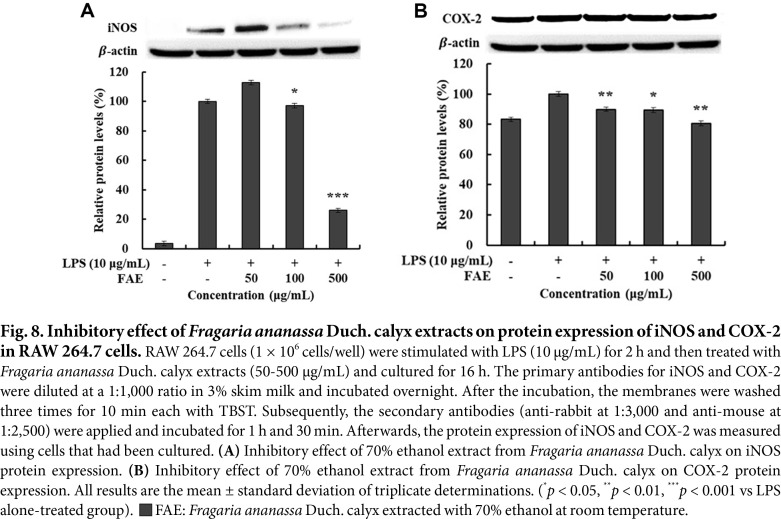
Fig. 8

The results of measuring protein expression of iNOS and COX-2 in 70% ethanol extracts from *Fragaria ananassa* Duch. calyx are shown in Fig. 8A-8B, where β-actin was used as a housekeeping gene. By measuring the protein expression of iNOS and COX-2 compared to the LPS single treated group, it was found that the 70% ethanol extracts from *Fragaria ananassa* Duch. calyx showed 73.98% and 19.44% inhibitory effects at 500 μg/mL.

### Measurement of the Effect of mRNA Expression Inhibition on iNOS and COX-2 via qRT-PCR

**Fig. 9:**
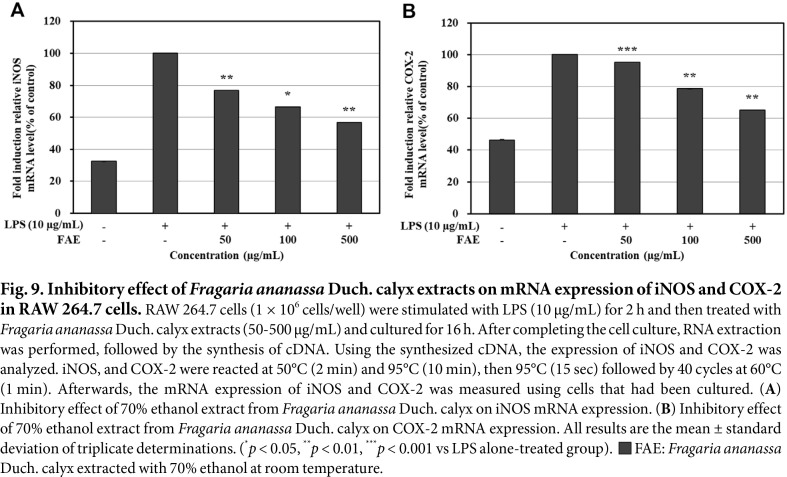
Fig. 9

To measure the inhibitory effect on the mRNA expression by iNOS and COX-2, the expression levels of each mRNA after treatment with 70% ethanol extracts from *Fragaria ananassa* Duch. calyx were measured, and the results are shown in Fig. 9A-9B. The difference in mRNA expression between the LPS single treated group and the LPS untreated group was confirmed, and the mRNA expression of iNOS and COX-2 showed 42.47% and 36.74%inhibitory activity of 70% ethanol extracts from *Fragaria ananassa* Duch. calyx at a concentration of 500 μg/mL, respectively.

### Measurement of the Effect of Inhibiting Phosphorylated MAPKs Protein Expression

Among the inflammatory response mechanisms, MAPKs, which consist of ERK, JNK, and p38, are involved in cell growth and differentiation and are known to regulate intracellular responses to stress and cytokine responses to various stimuli. These MAPKs are phosphorylated in LPS-stimulated macrophages and are involved in the regulation of pro-inflammatory iNOS, COX-2, and pro-inflammatory cytokine expression [48].

**Fig. 10:**
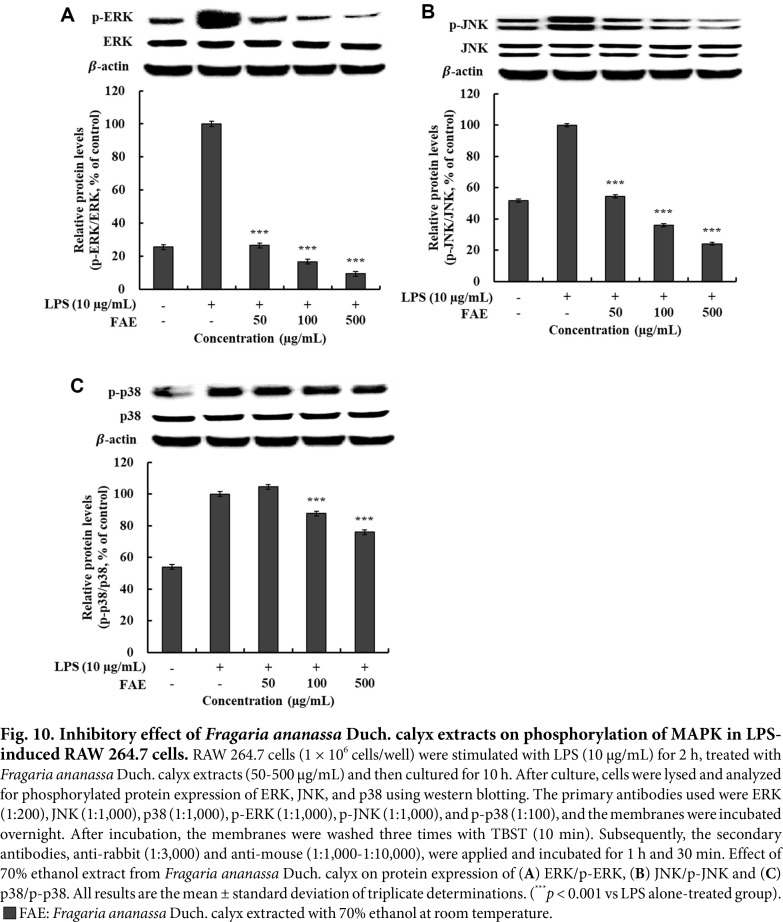
Fig. 10

The results of measuring the protein expression of phosphorylated MAPK factors p-ERK, p-JNK, and p-p38 by 70% ethanol extracts from *Fragaria ananassa* Duch. calyx are shown in Fig. 10A-10C. Compared to the LPS single treated group, 70% ethanol extracts from *Fragaria ananassa* Duch. calyx showed 90.53%, 50.21%, and 24.09%inhibitory effects on the protein expression of p-ERK, p-JNK, and p-p38 at 500 μg/mL.

### Measurement of the Effect of Inhibiting Phosphorylated NF-κB Protein Expression

NF-κB bound to IκB exists in an inactive form in the cytoplasm and is an important transcription factor. When stimulated by various inflammatory signals such as cytokines and pathogens, IκB is phosphorylated and degraded, allowing the NF-κB dimer (primarily p65) to translocate to the nucleus and initiate the transcription of target genes involved in the inflammatory response [49]. NF-κB activation through phosphorylation leads to the production of NO, PGE_2_, and pro-inflammatory cytokines. This process is crucial in the inflammatory response and has implications in various diseases [50].

**Fig. 11:**
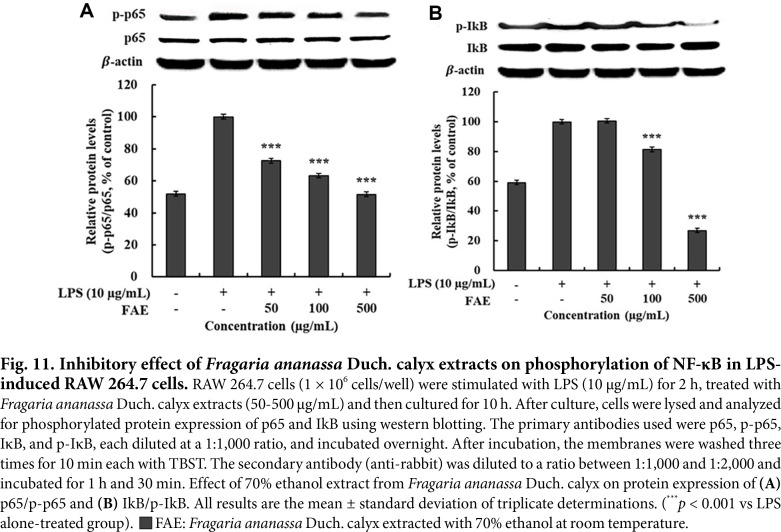
Fig. 11

The results of the protein expression of phosphorylated NF-κB, such as p-p65 and p-IkB, induced by 70%ethanol extracts from *Fragaria ananassa* Duch. calyx are shown in Fig. 11A-11B. Compared to the LPS-only treated group, the 70% ethanol extracts from *Fragaria ananassa* Duch. calyx at a concentration of 500 μg/mL inhibited the protein expression of p-p65 and p-IkB by 48.52% and 73.04%, respectively.

## Discussion

The berry portion of *Fragaria ananassa* Duch. is consumed largely as a fruit or as a component of processed foods such as fruit juice, yogurt, jelly, and jam [51]. This industrial processing is known to generate a significant amount of by-products (leaves, roots, and calyxes), which are typically disposed of as biomass wastes. These by-products of *Fragaria ananassa* Duch. contain high concentrations of phenolic compounds and can be used to obtain raw materials at low cost, in addition to lowering final disposal costs [18, 52]. The fruits, leaves, and roots of *Fragaria ananassa* Duch. have been studied for their various effects, including anti-inflammatory, antioxidant, anti-obesity, and apoptosis-preventing properties [53], but the calyx has been rarely studied. Previous studies have demonstrated that inflammatory factors such as NO, PGE_2_, iNOS, COX-2, TNF-α, and IL-6 produced through the MAPK and NF-κB pathways in LPS-stimulated RAW 264.7 cells contribute to the development of inflammatory diseases [54, 55]. Furthermore, the anti-inflammatory potential of berries, including black raspberries, blueberries, red raspberries, and blackberries, has been reported. These berries exert their effect by suppressing inflammatory mediators through the inhibition of MAPK and NF-κB signaling pathways, demonstrating significant anti-inflammatory activity [56, 57]. Therefore, to demonstrate the anti-inflammatory activity of the calyx among *Fragaria ananassa* Duch. by-products in LPS-induced inflammation in RAW 264.7 cells, this study verified the inhibitory effects on the production of inflammation-related factors NO, PGE_2_, iNOS, COX-2, TNF-α and IL-6, as well as the inhibition of MAPK and NF-κB signaling pathway.

Polyphenols are abundant in plants and are known to be powerful antioxidants and anti-inflammatory compounds that inhibit pro-inflammatory mediators and inflammation-related signaling pathways [58]. Before investigating its anti-inflammatory effect, the antioxidant properties of the 70% ethanol extracts from *Fragaria ananassa* Duch. calyx were investigated to determine if it inhibits oxidative reactions. The polyphenol content of the 70% ethanol extracts from *Fragaria ananassa* Duch. calyx was determined to be 265.86 ± 0.85 mg TAE/100 g.

Under normal conditions, free radicals are responsible for signaling and immunity in the body, but when they become excessive, they cause oxidative stress, which damages normal cells and tissues in the body, leading to inflammation [59, 60]. The 70% ethanol extracts from *Fragaria ananassa* Duch. calyx showed more than 89%EDA and 98% ABTS radical scavenging ability at 1,000 μg/mL. These results confirmed that the *Fragaria ananassa* Duch. calyx extract contains polyphenols, which are antioxidants and have been shown to be effective in scavenging free radicals.

Inflammation is a physiological defense that occurs in response to infection with an external stimuli such as LPS [61]. When this happens, various immune defense cells such as macrophages are involved and inflammatory factors are overexpressed, but if this reaction continues, the immune defense system is destroyed and diseases such as cancer occur [62]. A representative example of this inflammatory response occurs when macrophages recognize LPS, one of the pro-inflammatory mediators, through the formation of a TLR4 heterodimer, which induces the activation of intracellular transcription factors such as MAPK and NF-κB. These activated factors then translocate into the nucleus, where they promote the transcription of genes encoding inflammatory cytokines such as TNF-α and IL-6, as well as enzymes like iNOS and COX-2 [7, 63]. The marker of the inflammatory response, NO, is synthesized from L-arginine, an amino acid, by iNOS. The excessive generation of NO by iNOS is particularly implicated in chronic inflammatory diseases, where it perpetuates tissue injury and inflammatory signaling [64]. Additionally, when inflammation occurs due to infection or injury, arachidonic acid is released from cell membrane phospholipids and converted into PGE_2_ via COX-2. PGE_2_, a type of PG, plays a pivotal role in promoting inflammation, fever, and pain [65]. The expression of these mediators leads to the development of inflammatory diseases within the body [66].

Therefore, the current study found that the production of inflammation-related factors NO and PGE_2_ was inhibited by 70% ethanol extracts from *Fragaria ananassa* Duch. calyx in LPS-stimulated macrophages (RAW 264.7). This led to the assumption that the extract will also impact iNOS and COX-2 expression, which affect the regulation of NO and PGE_2_ production, as well as the production of pro-inflammatory cytokines TNF-α and IL-6, and thus the inhibitory effects on these were investigated. As a result, it was confirmed that the 70% ethanol extracts from *Fragaria ananassa* Duch. calyx significantly inhibit the protein and mRNA expression of iNOS and COX-2 and the production of pro-inflammatory cytokines.

The MAPK and NF-κB pathways are pivotal signaling mechanisms in inflammation and immune responses. The MAPK pathway includes three major kinases: ERK, JNK, and p38, which are activated by external stimuli such as LPS or pro-inflammatory cytokines [67]. Activation occurs via phosphorylation cascades, where upstream kinases like MAPK kinase activate MAPKs through phosphorylation. These MAPKs then translocate into the nucleus to activate transcription factors like Activator protein 1, driving the expression of pro-inflammatory genes, including TNF-α, IL-6, iNOS and COX-2. The MAPK pathway also plays a role in cell proliferation, differentiation, and apoptosis during inflammation [68]. The significant effect of 70% ethanol extracts from *Fragaria ananassa* Duch. calyx on p-ERK and p-JNK was confirmed, and although p-p38 did not have as significant effect as p-ERK and p-JNK, it showed an inhibitory effect of more than 20% at the final concentration, indicating that 70% ethanol extracts from *Fragaria ananassa* Duch. calyx was involved in the regulation of inflammatory response by attenuating the activation of MAPK signaling pathway and subsequently inhibiting inflammation-related factors.

The NF-κB pathway functions by regulating immune and inflammatory responses. It is kept inactive in the cytoplasm by IKK. Upon stimulation such as LPS binding to TLR4, the IKK complex phosphorylates IκB, marking it for degradation. This releases the p65 NF-κB dimers, allowing them to enter the nucleus and promote the transcription of inflammatory mediators such as IL-6, TNF- α, iNOS, and COX-2 [69, 70]. The 70% ethanol extract of *Fragaria ananassa* Duch. calyx was found to exhibit inhibitory effects on p-p65 and p-IκB, confirming its impact on the NF-κB signaling pathway. Based on this, this study is expected to be used as a basis for the association of inflammatory mediators and pro-inflammatory cytokines through the inhibition of MAPK and NF-κB signaling pathway by the *Fragaria ananassa* Duch. calyx extract.

Future research should focus on the isolation and characterization of active compounds from *Fragaria ananassa* Duch. calyx. Additional studies are needed to verify the anti-inflammatory effects of these isolated compounds and gain a deeper understanding of their potential applications. Based on the findings of this study, *Fragaria ananassa* Duch. calyx could be applied in various fields such as cosmetics and pharmaceuticals as an anti-inflammatory agent. If applied in pharmaceuticals, further animal testing would be required, while for cosmetics, animal testing is not permitted, necessitating the development of the ingredient for clinical trials. Thus, the foundational data from this study is considered highly valuable.

## Figures and Tables

**Table 1 T1:** Total polyphenol contents of extracts from *Fragaria ananassa* Duch. calyx.

Sample	Total polyphenol (mg TAE/100 g)
FAE	265.86 ± 0.85

All values are mean ± standard deviations of triplicate determinations. FAE, *Fragaria ananassa* Duch. calyx extracted with 70% ethanol at room temperature.
